# Maternal dietary supplementation with grape seed extract in reproductive hens increases fertility in females but decreases semen quality in males of the F1 generation

**DOI:** 10.1371/journal.pone.0246750

**Published:** 2021-02-25

**Authors:** Jérémy Grandhaye, François Lecompte, Pascal Chartrin, Maryse Leconte, Antonella Riva, Alix Barbe, Éric JeanPierre, Erika Caldas-Silveira, Patrice Ganier, Marine Chahnamian, Christelle Ramé, Joëlle Dupont, Pascal Froment

**Affiliations:** 1 INRAE Physiologie de la Reproduction et des Comportements (PRC) - UMR85 CNRS, IFCE, INRAE, Université de Tours, PRC, Nouzilly, France; 2 INRAE, UMR0083 Biologie des Oiseaux et Aviculture, Nouzilly, France; 3 INDENA, Tours, France; 4 INRAE - Unité Expérimentale du Pôle d’Expérimentation Avicole de Tours UEPEAT, 1295, Nouzilly, France; Universite Clermont Auvergne, FRANCE

## Abstract

Genetic selection in parental broiler breeders has increased their susceptibility to metabolic disorders and reproductive dysfunction. We have recently shown that maternal dietary grape seed extract (GSE) supplementation in hens improves fertility parameters, egg quality, oxidative stress in different tissues and the quality of F1 chicks. Here, we analysed the growth and fertility (both female and male) of the F1 generation animals and the quality of their offspring (F2 generation). Eggs issued from hens supplemented with GSE presented lower ROS production than control hens, suggesting a change in the embryonic environment. However, this did not affect the growth nor the body composition of male and female F1s from hatching to adulthood (37 weeks of age). At 37 weeks of age, the biochemistry analysis of the GSE-F1 muscle has revealed an increase in sensitivity to oxidative stress and a slight change in lipid composition. Both male and female F1-GSE groups presented a delay in puberty with a lower testis volume at 30 weeks of age and lower ovary development at 26 weeks of age. Adult GSE-F1 males did not present histological alterations of seminiferous tubules or semen production, but the semen quality was degraded due to higher oxidative stress and DNA-damaged spermatozoa compared with control F1 animals. In adult GSE-F1 females, despite the delay in puberty, the females laid more eggs of better quality (fewer broken eggs and a higher hatching rate). At hatching, the weight of the chicks from GSE-F1 females was reduced, and this effect was stronger in F2 male chicks (F2) compared with F2 control chicks (F2), because of the lower muscle volume. In conclusion, we can raise the hypothesis that maternal dietary GSE supplementation produces eggs with change in embryonic metabolism, which may affect in adulthood the fertility. The data obtained from the F1-GSE group pointed to a sex-specific modification with higher egg quality in females but semen sensitive to stress in males. Finally, male F2 chicks were leaner than control chicks. Thus, maternal dietary grape seed extract (GSE) supplementation in hens may impact on the fertility of the offspring in a sex-specific manner in subsequent generations.

## Introduction

Since the 1960s, the production of broilers has changed via intentional genetic selection to improve meat yield [1, 2]. In 2017, broiler meat production took first place in the world, with 118 million tons produced against 117 million of tons of pork, according to the Organisation for Economic Co-operation and Development (OECD) and Food and Agriculture Organisation (FAO). This choice of genetic manipulation to improve meat yield was accompanied by impaired cardiac performance, stress resistance, immunity and reproduction [3–6]. Genetic selection and ad libitum feeding cause an excess of energy [7], leading to aberrant follicular recruitment and dysregulation of preovulatory follicle selection, which induce multiple ovulations and lead to poor reproductive efficiency [8, 9]. This poor reproductive efficiency is linked to high levels of oxidative stress in animals [10, 11]. In order to improve fertility, dietary supplementation strategies with antioxidants have been used.

On farms, vitamin E and selenium are widely used in diet supplementation or in water to improve the oxidative status of animals. The use of selenium in the diet improved the activity of glutathione peroxidase in the liver, testes, sperm and seminal plasma [12]. In females’ diets, selenium limited the decrease in the laying rate [13], and supplementation of vitamin E improved fertility and hatching [14]. However, these molecules are not all of natural origin and are very dependent on the market price, which makes them expensive. Consequently, other sources of antioxidants are sought and in fact, researchers use plants and, specifically, polyphenols from plants.

Radwan et al. used thyme or rosemary at 1% in the diet for 3 months in laying hens. Adding these plants to the diet improved reproductive performance, egg weight and feed conversion in layer hens [15]. An experiment was conducted to evaluate the effect of two plants belonging to Chinese herbal medicines, *Ligustrum lucidum* and *Schisandra chinensis*, on laying performance during heat stress. Supplementation with a 1% extract of these two plants had ben eficial effects on egg production after heat stress (10% increase in egg production) [16]. Some studies showed an increase in the activity of antioxidant enzymes in the livers of laying hens when an antioxidant diet was used [17, 18]. The liver is the source of yolk formation, and Aleksandro Da Silva’s team has shown that antioxidant supplementation in the diets of laying hens improved the quality and antioxidant status of eggs [19–21]. Moreover, a study used resveratrol (a grape polyphenol) supplementation in the diets of laying hens. This improved the quality of the eggs (improved albumin viscosity and protein content) and decreased the cholesterol content of the yolk [22]. In mice, a resveratrol enriched diet can maintain a new phenotype across generations induced by transgenerational inheritance mechanism [23, 24]. Modification of the global DNA methylation in F1 and F2 generation was associated to change of epigenetic enzyme expression, raising the hypothesis that maternal bioactive compound diet can change the metabolism and others phenotype through generations.

The modifications of egg components by maternal diet could have an impact on offspring growth; however, the effects on the offspring have rarely been studied. An example of a study focusing on offspring described that 3% carotenoids in the diets of parental hens improved the antioxidant rate in the offspring’s livers during the first 2 weeks of life [25]. Moreover, when the hens were supplemented with selenium, the offspring showed an improvement of tissue glutathione peroxidase activity [26]. Maternal dietary supplementation with GSE increased the live body weight and viability of chicks at hatching and 10 days of age [27]. However, these authors studied only the first weeks of life of the progeny, and data on the adult stage as well as on reproductive performance were frequently missing.

In hens, dietary GSE supplementation has been demonstrated to improve the fatty acid profile in the liver [28], stimulate the immune system [29] and slow the parental ovarian aging process [30]. However, the effects of grape seed extracts on the next generation exposed during the embryonic period were not examined. The objective of the present study was to investigate in broiler progeny the effects of maternal dietary supplementation with GSE on growth and reproductive performance by using non-invasive imaging tools.

## Materials and methods

### Animals

Forty female and 40 male chicks (F1) were studied from birth to 37 weeks of age. All chicks (F1) were issue from two groups of 20 female parental Cobb 500 broiler breeders (F0) obtained from the chicken breed selection farm (commercial breeding unit of Hendrix Genetics, Saint Laurent de la Plaine, France). The first group received a control diet and the second, the same diet supplemented with GSE at 1% of the total diet from their birth. At the 30th week, female were inseminated artificially with a pool of semen of 8 cocks receiving a control diet (Cobb500, same breed selection farm). The GSE supplement was provided by INDENA (Tours, France) and was prepared from white wine production. The GSE present a relatively low amount monomers forms (catechin and epicatechin, between 5.0% to 15.0%) and a high contents of oligomeric proanthocyanidins (≥90.0%) as determined by HPLC (High Performance Liquid Chromatography) by INDENA. as detailed in previous studies [27, 31]. Then, the study was composed of four groups (F1): male (n = 20) and female (n = 20) chicks exposed *in ovo* to yolk modified by maternal GSE dietary supplementation (GSE) and male (n = 20) and female (n = 20) control chicks.

### Breeding

Each group was divided into five pens, each with an area 3 m^2^ (four chicks/pen). The animals were reared in room initially bedded with 8 cm of clean shavings or hulls at the Experimental Unit of the Poultry Experimentation Center of Tours (UEPEAT, Nouzilly, France), according to conventional breeding conditions. The lighting conditions were 14 h of light per day (minimum light intensity of 20 lux) at their arrival, followed by a gradual decrease to approximately 8 h in the first week, kept constant until the age of photostimulation (21st week) and then a gradual increase to 14 h of light per day at the end of the study (37th week) every 4 weeks. The housing temperature began at 31 °C and was decreased gradually to 21.5 °C, with humidity ranging between 35% and 70% during the 37 weeks of the study. Each chick was weighed weekly. From the first day to the fourth week, all chicks (n = 80) received an ad libitum diet (free access to food). From the fourth week to the 37th week of age, chicks received three different diets according to Hendrix Genetics’ recommendations: grower (weeks 4 to 18), prebreeder (weeks 18 to 21) and breeder diets (weeks 21 to 37). In order to adjust the amount of feed consumed by animals, animals in the control pens were weighed, and then feed was adjusted weekly with respect to the theoretical curve provided by the supplier (S1 Fig).

All experiments were approved by the Ethics Committee in Animal Experimentation of Val de Loire CEEA Vdl and registered by the National Committee ‘Comité National de Réflexion Ethique sur l’Expérimentation Animale’ under number 19 (APAFIS# 10237-201706151202940v3). All experiments were performed in accordance with the European Communities Council Directive 2010/63/UE.

### Male fertility

Male fertility parameters were evaluated by using ten 35-week-old roosters to measure the semen concentration and the effect on mass motility. For that, males were given preliminary training for 1 month to enable them produce enough semen. The back-lumbar massage method was adopted in this study to collect the semen [32].

Briefly, mass sperm motility was assessed by analysing a drop of semen, which was deposited on a pre-warmed glass slide (≈37 °C), and the edge of the drop was observed at low magnification (objective 10×) on the thermally controlled stage of a phase-contrast microscope. Observations at the edge of the semen drop provided an assessment of the type and intensity of sperm wave and swirl movements, which was termed as wave motion or mass sperm motility. This mass sperm motility was scored subjectively from 0 (no motion) to 8 (numerous rapid waves) on a scale with steps equal to 1 and was perfomed always by the same observer.

### Female fertility

At the 23rd week of age, all eggs were collected and individually weighed (KERN Scale—ref. PCB 250–3, Kern and Sohn, Balingen, Germany). Broken eggs or double-yolk eggs were noted. Eggs were analysed with an Egg Tester (Egg Tester, Orka Food Technology), where the following parameters were obtained: STZ: egg resistance (Newton), HU: Haugh unit, YF: Yolk colour between 1 and 16 according to the DSM (formerly Roche), YH: yolk height (mm), YD: yolk diameter (mm), YI: yolk index (yolk index as a ratio of the yolk height to its diameter; and yolk weight), THK: shell thickness (mm), dried shell weight (g), albumen volume (mL), pH of albumen, egg shape index (calculated as the ratio of egg width to egg length), shell weight, shell thickness, albumen (weight, height and Haugh unit).

Egg fertility was assayed by artificially inseminating hens with 2 × 10^8^ spermatozoa from control roosters on the first and second days of the 27^th^ week. Eggs from each hen were collected and incubated every 7 days. The number of fertilized eggs was evaluated by candling after 7 and 14 days of incubation. After candling at 7 days, eggs without clear viable embryos were opened to determine whether they contained an early dead embryo or an unfertilized oocyte. The percentage of dead embryos/incubated egg and the hatching percentage were measured. The bodyweight and growth gain were measured during the first 12 days of life in viable chicks (F2).

At 37 weeks, all animals were stunned by electrocution and killed by exsanguination as recommended by the ethical committee. The blood samples were collected in heparin tubes, centrifuged to obtain the plasma (1500 g, 10 min, 4 °C), aliquoted and stored at −20 °C until analysis. The testes, ovaries, follicles F1 and muscle were weighed and stored at −80 °C until analysis.

### Computerized tomography (CT-scan) analysis

Chickens (F1) were anesthetized for 2 hours, then blood samples were collected at the occipital sinus in heparin tubes, and the body composition of all animals was analysed by computerized tomography (CT-scan, Siemens Somatom Definition AS, Siemens Corp., Germany) each month from 8 weeks to 37 weeks of age.

Body composition was determined as previously described in [33]. For illustration purposes, the window level for pixel density measurement was (20; 80 HU) for the pectoralis major muscle, (-90; 30 HU) for fat, (150; 2500 HU) for total cortical bone (850; 2000 HU) and (0; 80) for testis. The length of the right tibia was measured in adult chickens (F1) and chicks (F2).

### Oxidative stress

The ROS-Glo^™^ H_2_O_2_ Assay (Promega, Charbonnieres, France) was used to analyse oxidative stress in the yolk, pectoralis major muscle and semen. Assays were performed according to the manufacturer’s instructions. Briefly, after treatment, samples were stressed with H_2_O_2_ Substrate Solution for 3 h, then incubated for 20 min with ROS-Glo^™^ Detection Solution in the dark to stabilize the luminescent signal. The plate was measured using a Luminoskan Ascent microplate reader (VWR International, France) to record luminescence.

GSH concentrations in muscle tissue were measured by using the GSH Kit (Promega, Charbonnieres, France), according to the manufacturer’s instructions.

### Metabolites

ATP concentrations were measured by using the CellTiter-Glo^™^ ATP Assay Kit (Promega, Charbonnieres, France). Glycogen assay kits (Cell Biolabs, INC., San Diego, USA) was used to quantify the glycogen content of the pectoralis major muscle. Assays were performed according to the manufacturer’s instructions.

### Fatty acid composition

The total lipid content and fatty acid composition were determined in the pectoralis major muscle by gas chromatography as previously described in [34]. Briefly, lipids were extracted gravimetrically in methanol:chloroform (1:2), and the fatty acid composition was determined by gas chromatography (Autosystem; Perkin Elmer, St Quentin en Yvelines, France) after transmethylation of lipids. Methyl esters were identified and quantified by comparison with standards (Sigma-Aldrich, l’Isle d’Abeau Chesnes, France).

### Immunofluorescence

Spermatozoa were fixed with PAF 4% for 15 min at room temperature and deposited on a glass slide. Then, fixed spermatozoa were incubated with PBS 1X / 0.1 M glycine for 15 min at room temperature to saturate the aldehyde groups and permeabilized with 0.1% Triton X-100 (w/v) in PBS for 15 min, and nonspecific binding sites were blocked in 2% Bovine Serum Albumin (BSA)/PBS for 15 min. Cells were incubated for 60 min at room temperature with the following primary monoclonal antibodies against DNA damage diluted at 1:100 in 1% BSA/PBS (Sigma-Aldrich, l’Isle d’Abeau Chesnes, France). Mouse IgG (Sigma-Aldrich, l’Isle d’Abeau Chesnes, France), was used as a negative control. After the incubation, spermatozoa were washed three times in PBS and were incubated for 45 minutes at room temperature with a goat anti-mouse IgG Alexa Fluor^®^ 488 antibodies (diluted at 1:500 in 1% BSA/PBS). Spermatozoa were counterstained with 4’,6’-diamidino-2-phenylindole (DAPI), and mounted on glass slides with Fluoroshield mounting medium (Sigma-Aldrich, l’Isle d’Abeau Chesnes, France) and examined using standard immunofluorescence microscopy. Staining was quantified by using the software ImageJ (NIH, Bethesda, Maryland, USA) on at least 500 spermatozoa per animal (three animals per condition).

Analysis of mitochondrial activity and oxidative stress was performed on fresh semen with, respectively, MitoTracker (Invitrogen Carlsbad, USA) and CellROX staining reagent (Invitrogen Carlsbad, USA), incubated for 30 min at 37 °C. Then, spermatozoa were analysed by flow cytometry (MoFlo Astrios^EQ^, Beckman Coulter, USA). Twenty thousand spermatozoa were analysed per condition and per sample (five animals per condition).

### Histology

Testes embedded in paraffin were serially sectioned to a slice thickness of 7 μm. Deparaffinized sections were hydrated and washed in a PBS bath for 5 min. Then, sections were stained with a haematoxylin-eosin solution (Sigma-Aldrich, l’Isle d’Abeau Chesnes, France), and the diameter of the round or nearly round transverse section of seminiferous tubule was measured for each testis using the software ImageJ (NIH, Bethesda, Maryland, USA) (*n* = 30 measurements per animal, 10 animals per group).

### Statistical analysis

Data were assessed for normality using a Shapiro-Wilk test normality test. All data passed normality test (alpha = 0.05).

The differences for the following parameters were analyzed using the unpaired Student t test (GSH, ROS, Glycogen, ATP content; testis and F1 follicle weight; diameter of seminiferous tubule; semen concentration and volume; percentage of DNA damage, cellrox positive spermatozoa; number of egg; percentage of broken eggs and embryonic mortality; volume of different F2 chick tissue.

For the following data performed at different time (mainly by CT-Scan analysis), we have used an ANOVA method with a mixed effect model in repeated measures. We have observed a "time effect" for all following data. To identify the effect of GSE, we performed an ANOVA with Tukey’s multiple comparison test in order to determine whether the means of the following factor were significantly different between the 2 groups for each time. Factors analysed were: Bodyweight, egg weight; fat content in pectoralis muscle; pectoralis, testis volume; number of yellow follicle and ovarian fat tissue.

Data were analyzed using GraphPad Prism version 8.0.2 for Windows, GraphPad Software, San Diego, California USA, www.graphpad.com.

The results are expressed as mean ± SEM. Values were determined to be significant to control when *p < 0.05, **p < 0.01, ***p < 0.001.

## Results

### Growth performance

**Fig 1:**
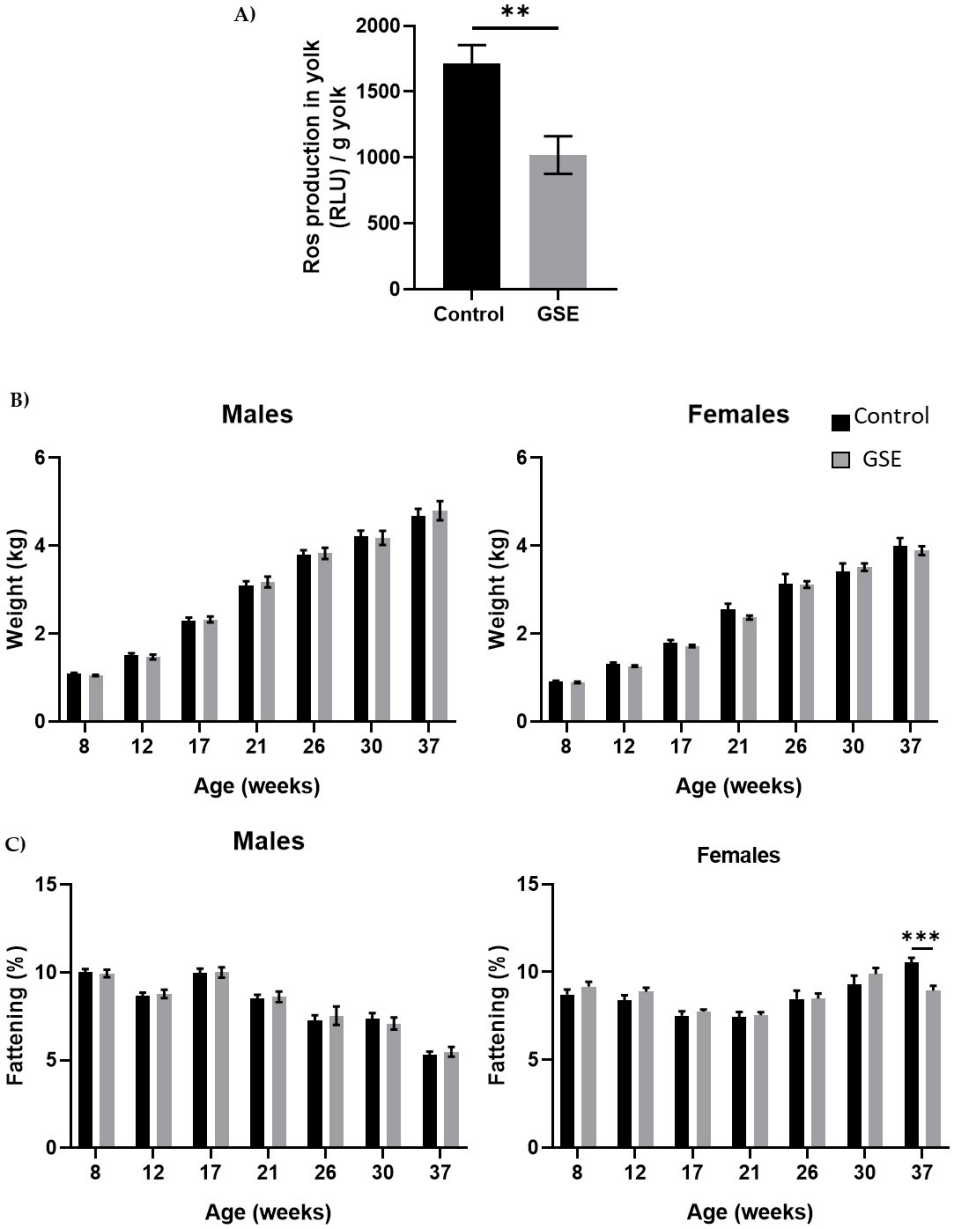
Growth curve. **(A**) Level of Reactive Oxygen Species (ROS, H_2_0_2_) in egg yolk of broiler hens fed 1% GSE dietary supplementation (n = 10 egg yolks analysed per group). Results are expressed in Relative Luminescent Units per gram of yolk. **(B)** Live body weight of males (left panel) and females (right panel) from 8 to 37 weeks of age (n = 20). Animals were weighed every 4 weeks. **(C)** Fattening level in male (left panel) and female (right panel) broilers was assessed by CT scan from 8 to 37 weeks of age (n = 10 animals/sex). Results are presented as means ± SEM. *P* values were considered significant if **, p < 0.01. ***, p < 0.001.

**Fig 2:**
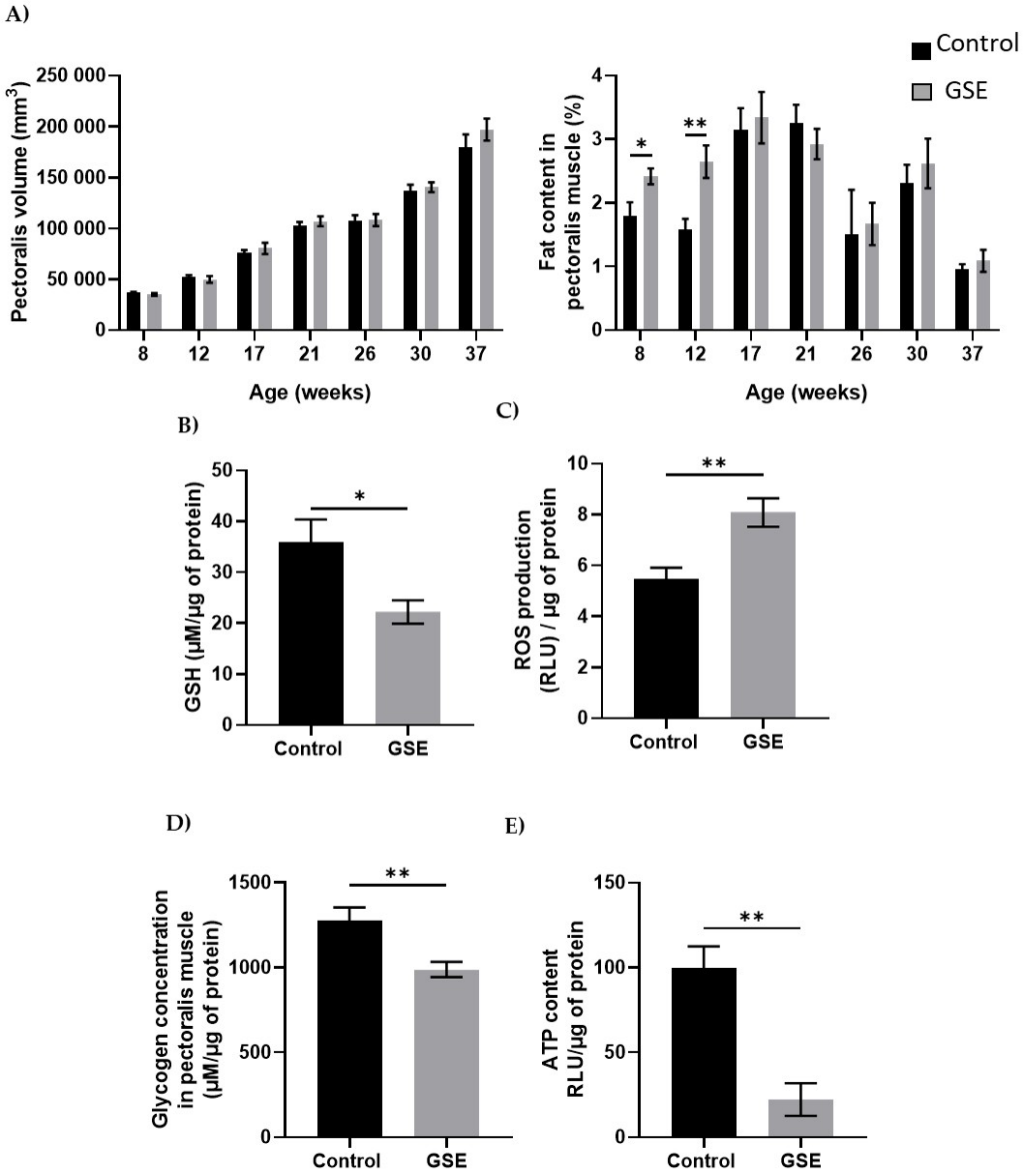
Muscle development. **(A)** Volume of a section of the right pectoralis muscle (left panel) and percentage muscle fat content (right panel) in male chickens was measured from weeks 8 to 37 (n = 10 animals) was assessed by CT scan every month. **(B**) Level of Reactive Oxygen Species (ROS, H_2_0_2_, expressed in Relative Luminescent Units/μg of protein) and GSH content (μM/μg of protein) **(C**) was measured in muscle (n = 5 per group). **(D)** Glycogen (μM/μg of protein) **(E**) and ATP content were measured in the pectoralis muscle (n = 5 per group). Results are presented as means ± SEM. *, p < 0.05. **, p < 0.01.

The content of fertilized eggs from mothers who received a diet enriched with 1% grape seed extract (GSE) showed lower reactive oxygen species production than eggs from control mothers. The embryonic environment is modified in the eggs of GSE mothers compared with control mothers (Fig 1A, p < 0.01). However, the growth performance of the chicks issued from GSE-mothers did not show any difference until the age of 37 weeks, compared with the control animals (Fig 1B). We performed a kinetic analysis of fatness, bone and muscle parameters until 37 weeks of age (Figs 1C and 2, S2 Fig). Likewise, the level of fattening measured by CT scan was similar except at 37 weeks of age, during the laying period, with a lower percentage of fat in F1-GSE females (Fig 1C, p < 0.001). F1 males and females are denoted as “F1-GSE” until the end of the study. The bone volume was similar between groups but was composed of a higher cortical percentage in the tibia in the F1-GSE group. The F1-GSE tibia presented higher fracture resistance than the control tibia (S2 Fig).

### Muscle quality

In the male, the change of environment *in ovo* did not affect the development of the pectoralis major nor the percentage of fat (Fig 2A). However, we have noted a modification of the distribution of fatty acids with an increase in saturated fatty acids (SFA) of 2% and palmitic acid (C16) and a decrease of about 4% in monounsaturated fatty acids (MUFA) between the F1-GSE group and the control group (Table 1, p < 0.05). In addition, at 37 weeks of age, the GSH content was reduced and was associated with an increase in the production of reactive oxygen species (Fig 2B and 2C, p < 0.05). The muscle content of glycogen and ATP was lower in the F1-GSE group (Fig 2D and 2E).

**Table 1:** Fatty acid composition of pectoralis major.

Fatty acids	Control	GSE
Lipid content	0,94 ± 0,08	0,92 ± 0,07
Myristic acid, C14:0	0,35 ± 0,03	0,31 ± 0,03
Myristoleic acid, C14:1	0,21 ± 0,06	0,12 ± 0,02
Palmitic acid, C16:0	3,88 ± 0,41^a^	5,30 ± 0,47^b^
Palmitoleic acid, C16:1	25,71 ± 0,88	23,77 ± 0,62
Stearic acid, C18:0	11,88 ± 0,55	12,78 ± 0,42
Oleic acid, C18:1	26,65 ± 0,89	24,33 ± 0,93
Linoleic acid, C18:2 n-6	19,91 ± 1,28	19,91 ± 0,62
Linolenic acid, C18:3 n-3	0,68 ± 0,09	0,61 ± 0,07
Arachidic acid, C20:0	0,09 ± 0,01	0,12 ± 0,02
Eicosenoic acid, C20:1	0,18 ± 0,01	0,18 ± 0,01
EPA, C20:5 n-3	8,15 ± 0,81	9,84 ± 0,85
Adrenic acid, C22:4 n-6	0,60 ± 0,07	0,64 ± 0,05
DPA, C22:5 n-3	0,92 ± 0,11	1,19 ± 0,13
DHA, C22:6 n-3	0,79 ± 0,07	0,92 ± 0,08
SFA	16,20 ± 0,88^a^	18,51 ± 0,76^b^
MUFA	52,75 ± 1,49^a^	48,39 ± 1,27^b^
PUFA	31,04 ± 1,42	33,10 ± 0,76
n-6 FA	20,51 ± 1,30	20,54 ± 0,64
n-3 FA	10,53 ± 0,86	12,56 ± 0,90
n-6 FA/n-3 FA	2,19 ± 0,24	1,82 ± 0,14

Percent lipid content and fatty acid composition (percentage of total fatty acids) of pectoralis major (mean ± SE, n = 12). Different individual letters (a, b) indicate a significant difference (p < 0.05) between groups. FA, fatty acid; EPA, eicosapentaenoic acid; DPA, docosapentaenoic acid; DHA, docosahexaenoic acid; SFA, saturated fatty acids; MUFA, monounsaturated fatty acids; PUFA, polyunsaturated fatty acids.

### Male reproductive performance

**Fig 3:**
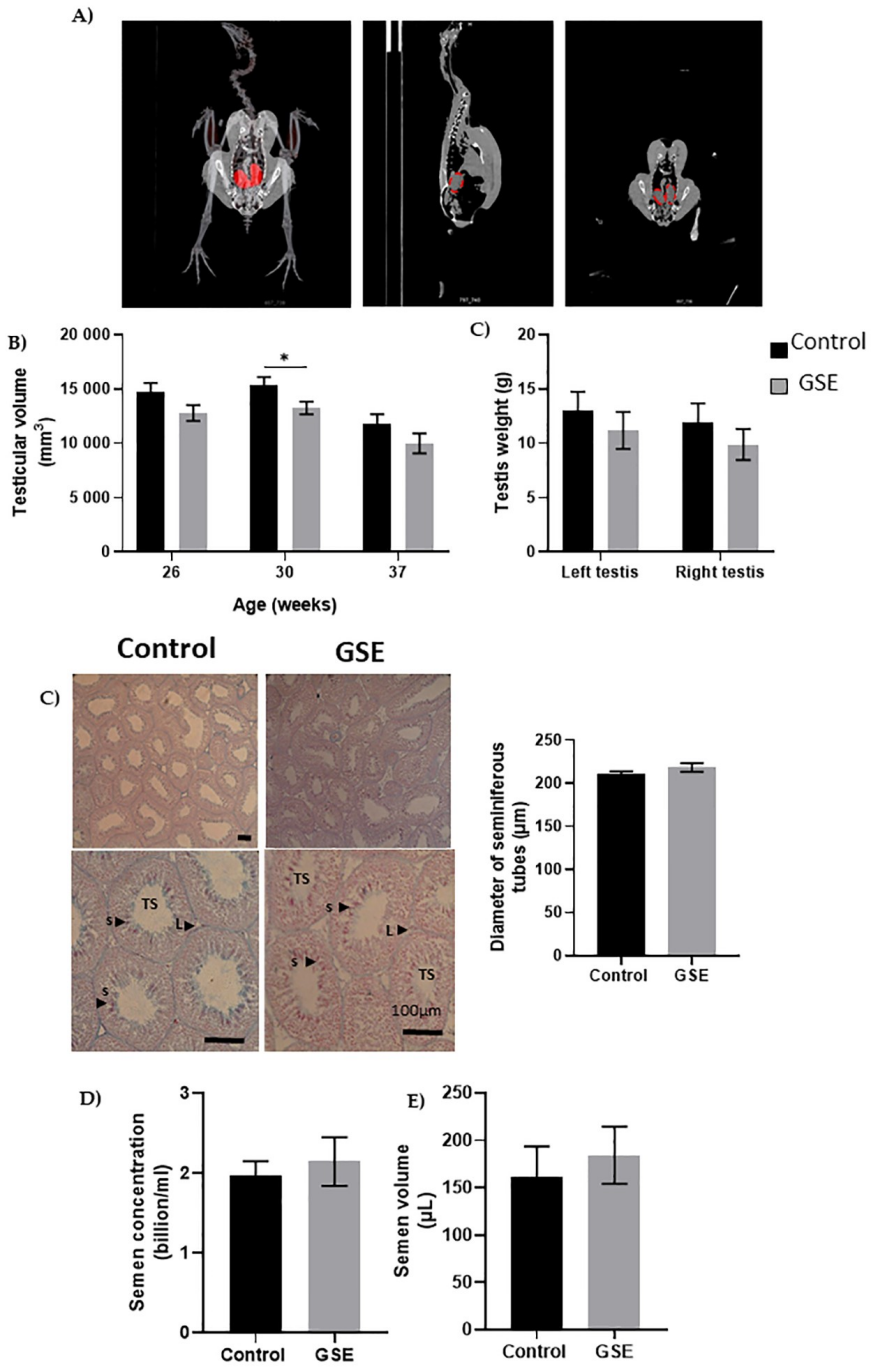
Testicular histology. (**A**) Representative picture of computerized tomography analysis of testes (red). **(B)** Volume of testes from weeks 26 to 37 (n = 10 animals) **(C**) Testis weight at 37 weeks of age. **(C)** Representative picture of seminiferous tubule histology of 37-week-old broilers. TS, seminiferous tubules composed of germ cells and Sertoli cells; L Leydig cells; s spermatozoa. The diameter of seminiferous tubules (μm) is represented to the right (n = 10 per group). **(D)** Semen concentration (billion/ml) (n = 10 per group) and **(E)** semen volume (μL) is represented in both groups (n = 10 per group). Results are presented as means ± SEM. *, *p < 0*.*05*; ***, *p < 0*.*001*.

**Fig 4:**
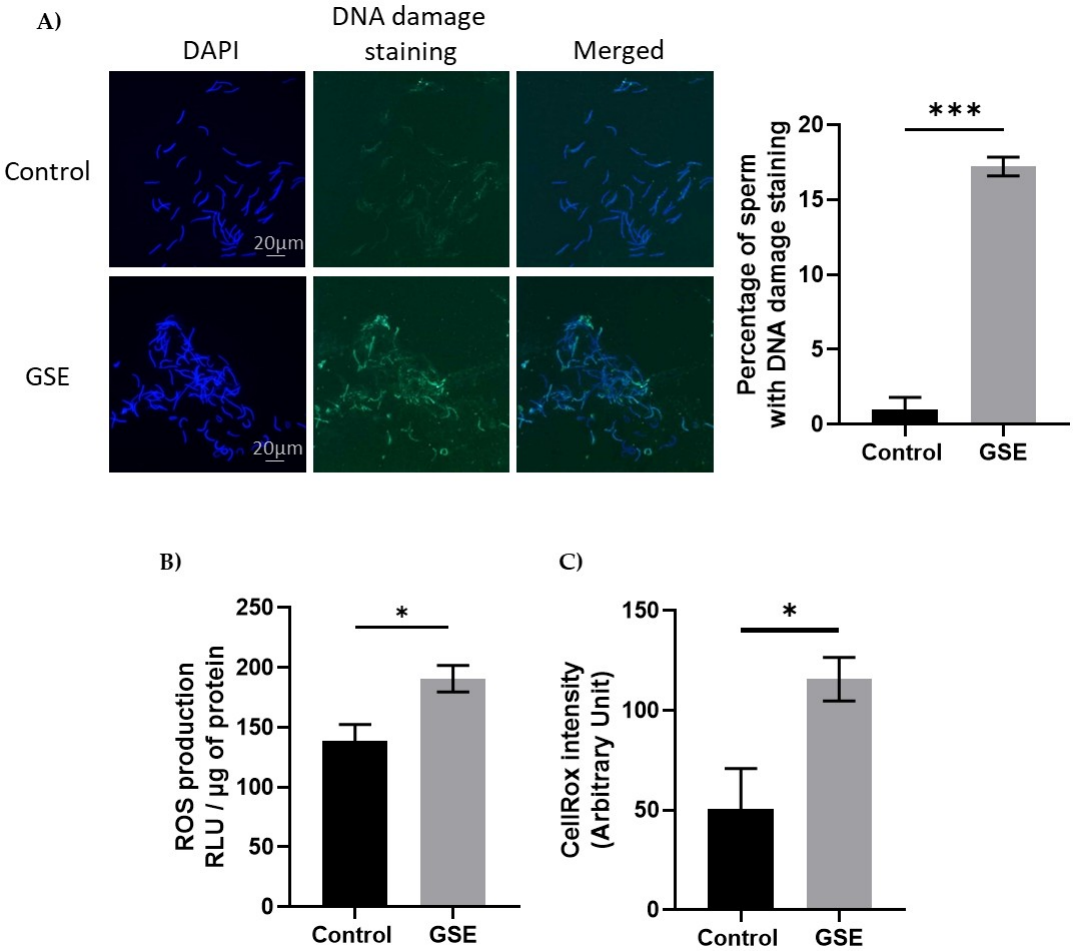
Marker of sperm quality. **(A**) Representative picture of DNA damage staining on spermatozoa from control or F1-GSE group (200 spermatozoa/animal with n = 5 animals per group) (**B**) Level of Reactive Oxygen Species (ROS, H_2_0_2_) in spermatozoa (n = 7 per group) and **(C)** quantification of oxidative stress staining was analysed by CellROX staining. Intensity was quantified using ImageJ (Arbitrary units) (200 spermatozoa/animal with n = 5 animals per group). Results are presented as means ± SEM. *, p < 0.05. ***, p < 0.001.

We evaluated fertility performance in both sexes, in particular, gonadal development using CT scanning of the same animals. We noticed a delay in gonadal development in both sexes in F1-GSE compared to control animals. In males, testicular volume was reduced at 26 (p = 0.07) and 30 weeks of age (p < 0.05). At 37 weeks, the testes of the F1-GSE animals again became similar to the controls; although they appeared slightly lower in volume and weight, the difference was not significant (Fig 3A–3C). Testicular histology did not show any alteration between the two groups (Fig 3C) nor any difference in the production of spermatozoa (Fig 3D and 3E). The mass motility (mass sperm motility score: F1-control: 2.34+/- 0.31 versus F1-GSE: 2.38+/-0.33, p > 0.05, n = 20 per group) and the mitochondrial activity measured by MitoTracker staining (F1-control: 98.76+/- 0.64% versus F1-GSE: 96.06+/- 1.48% MitoTracker-positive spermatozoa, p > 0.05, n = 5 per group) were identical between groups. However, sperm quality appeared to be affected, since spermatozoa from F1-GSE animals had more DNA damage (Fig 4A) and a higher ROS content (ROS production, p < 0.05; and CellROX staining intensity, p < 0.001) (Fig 4B and 4C).

### Female reproductive performance

**Fig 5:**
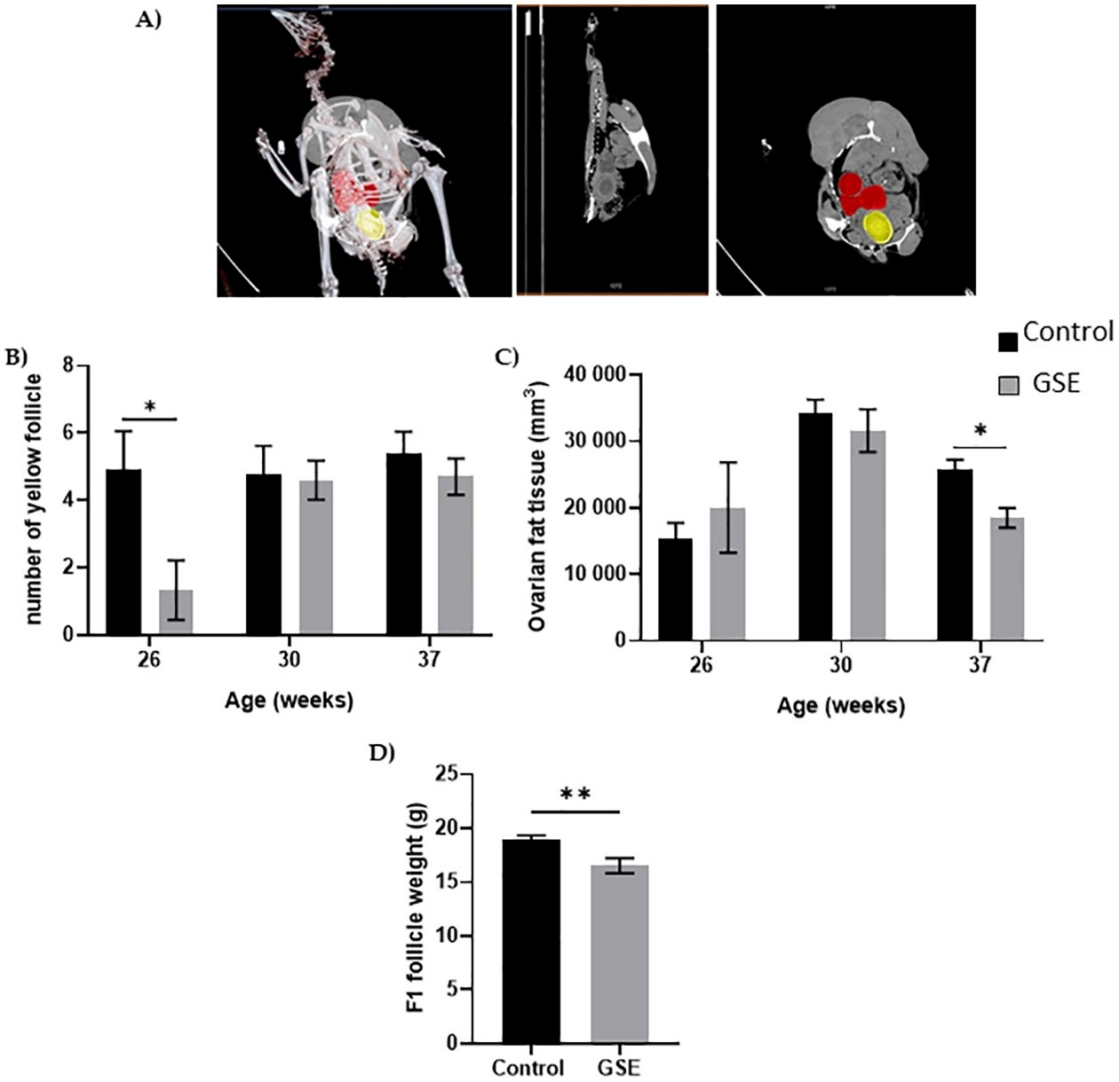
Ovarian anatomy. (**A**) Representative picture of computerized tomography analysis of yellow follicles (red) and egg formation (yellow) (n = 10 per group). **(B)** Number of yellow follicles; **(C)** volume of the ovarian fat analysed by computed tomography; **(D)** the weight of preovulatory follicles in the F1 was measured at 37 week of age (n = 10 per group). Results are presented as means ± SEM. *, p < 0.05. **, p < 0.01.

**Fig 6:**
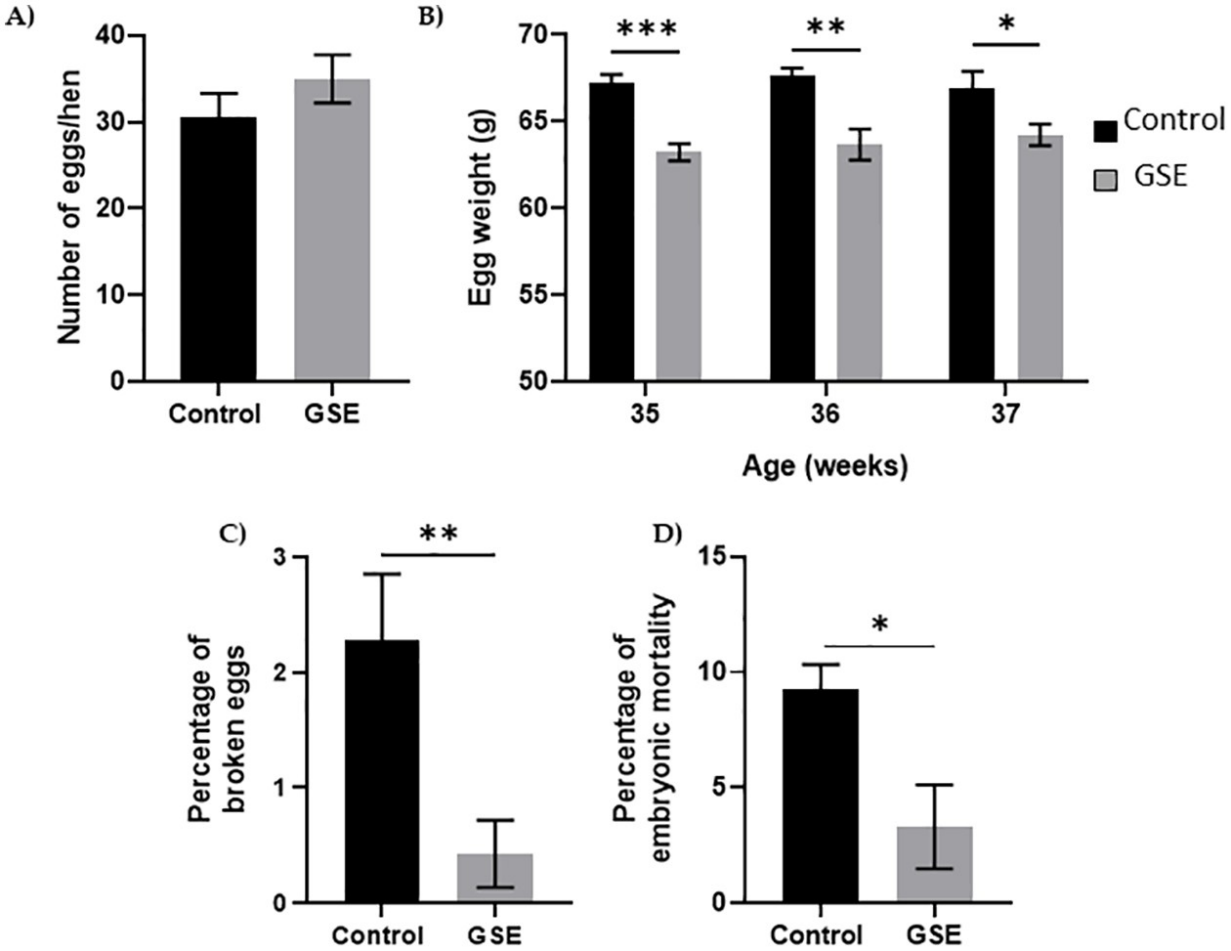
Reproductive performance. (**A**) Cumulative egg number during a 60-day period (29-37-week- old hens) (n = 20 per group), **(B)** Kinetics of egg weight during the reproductive period, **(C)** Percentage of broken eggs, **(D)** Percentage of embryonic mortality in incubated egg was measured in 200 eggs from 20 hens per group. Results are presented as means ± SEM. *, p < 0.05. **, p < 0.01. ***, p < 0.001.

**Fig 7:**
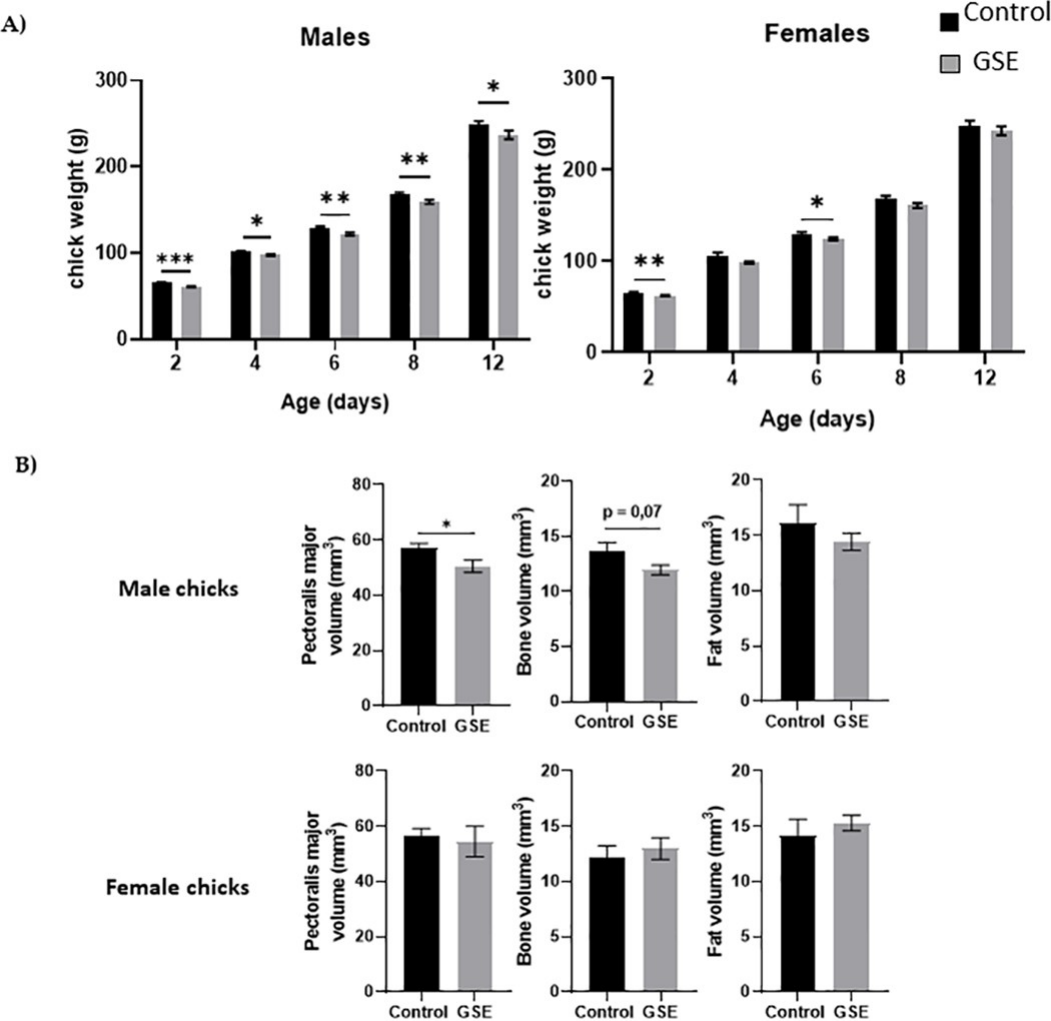
Body composition of the F2 chicks. (**A**) Body weight (g) of male and female chicks (F2) at hatching (D0) and 12 days of age (D12) from F1 control and F1-GSE hens (n = 60). (**B**) The pectoralis major muscle, fat and bone volume of male and female chicks were measured at day 12 of age (n = 10 animals/sex) by computed tomography. Results are presented as means ± SEM. *, p < 0.05. **, p < 0.01. **, p < 0.01.

In females, the number of yellow follicles measurable at 26 weeks of age was markedly lower in F1-GSE than in controls (Fig 5A and 5B, p < 0.05). The fat volume in the ovary area was reduced in F1-GSE animals at 37 weeks of age (Fig 5C, p < 0.05), associated with a lower weight (Fig 5D, p < 0.01). The two groups of females were fertilized with control semen. The number of eggs laid in 60 days was 30.5 eggs/hen in the controls and 35 eggs/hen in the F1-GSE group (Fig 6A). The eggs presented a lower weight in the F1-GSE females (Fig 6B) and a smaller albumen volume (S1 Table). The other egg parameters were unaffected by group status (S1 Table). However, the quality of eggs was increased, with an extremely low number of broken eggs (Fig 6C, p < 0.01), lower embryonic mortality and an improved hatching rate (Fig 6D, p < 0.05). The weight of the chicks (F2) from the F1-GSE females was slightly lower until 12 days of age (Fig 7A). This phenotype mainly affects the male sex and is associated with a limited muscle volume (Fig 7B). No difference in food intake was noted between groups (25.48+/- 4.14 g food intake/day/chick F2-control, n = 137 versus 26.30+/- 4.59 food intake/day/chick F2-GSE, n = 141, p > 0.05).

## Discussion

**Fig 8:**
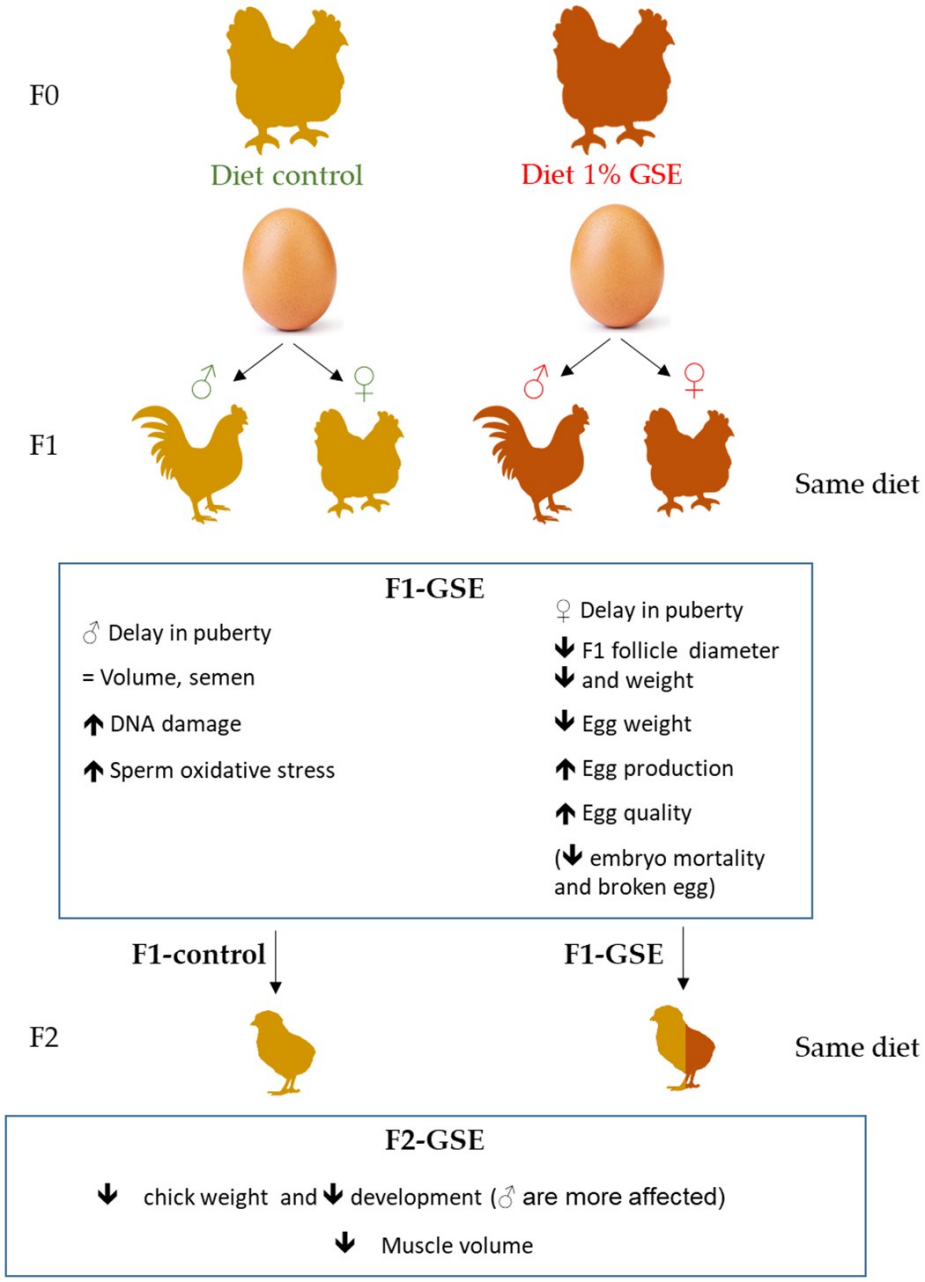
Consequence of maternal GSE supplementation on offspring fertility schematic summary of the consequences on cell metabolism, steroid production and cell proliferation.

The objective of the present study was to evaluate the generational effects of maternal exposure to GSE in the diet on physiological parameters of offspring chicken during growth and the reproductive period (Fig 8). The growth physiological and metabolic parameters evaluated, demonstrated that GSE addition to the parents’ food influenced the muscle metabolism of offspring and modified their reproductive performance. Hence, consequences on offspring could be due to a change in the egg components content modified by the maternal GSE intake such as vitamins, lipids or polyphenol content.

Manipulation of the diets of breeding females is commonly used without necessarily considering the consequences for the offspring. New approaches to agroecology or the improvement of fertility and the well-being of breeders involve using food supplements containing plant extracts. One such example is grape seed extract, which has antioxidant properties. In a previous study, maternal dietary supplementation with GSE was shown to increase offspring viability and reduce oxidative stress, suggesting a beneficial effect on reproductive performance [27]. It is important to know the consequences of this modification of the environment in which the embryo develops. In this study, we observed that growth was not impacted. However, for some years, studies have shown the direct effect of dietary GSE on the performance of broilers. The use of 5 g/kg of GSE from hatching to 21 days of age slowed the growth rate and lowered the feed conversion ratio [35]. Supplementation at 10 g/kg from hatching to 40 weeks of age did not affect food intake and fattening but decreased body weight [27].

In the same experiment, we showed an increase in the antioxidant status of animals and a reduction in ROS in egg yolk when hens were fed with 10 g/kg of GSE [31]. We confirmed this lower ROS production, suggesting that the environment was altered in eggs from hens fed with GSE. Previous studies have shown that the environment modulates and influences the physiological and morphological development of embryos, leading to effects on the development of the chick’s phenotype [36]. Parental diet and egg composition can alter offspring’s behaviour, performance or even vulnerability to disease [37].

We have confirmed that maternal GSE supplementation can affect lipid and glucose metabolism in broiler offspring. We have focused in the pectoralis major muscle of F1-GSE, a metabolic tissue, where fatty acid profile and glycogen content are also associated to the index of meat quality. We have observed in muscle, that the fatty acid proportion was slightly affected, with a decrease in monounsaturated fatty acids (MUFA) associated to an increase in saturated fatty acid (SFA) levels. Glycogen content was lower in F1-GSE and was associated to a decrease in ATP content, the energy stored in the muscle. These data suggest a breakdown of muscle glycogen, which is the first step in glycolysis [38]. In addition, we have noted a higher oxidative status in F1-GSE chickens. Indeed, in the present study, we have reported a decrease in GSH and an elevated oxidative ROS content in muscle. A low GSH content is frequently associated with oxidative stress [39, 40]. Increased levels of oxidative stress can alter DNA and RNA structures and gene expression, damage the cellular membrane and modify protein function [41–44].

We speculated that if metabolism is affected, other closely related functions, such as fertility, must also be affected. Some reproductive parameters were studied during the experiment; both males and females presented growth retardation of the gonads at puberty in F1-GSE animals. In addition, only 30% of GSE-F1 females ovulated at 21 weeks of age, compared with 70% of control females. This showed a delay in the onset of puberty in these females as in the males. However, this retardation disappeared at 37 weeks of age, when the volumes of the treatment and control groups equalized (i.e. diameter of seminiferous tubes, volume and concentration of semen in males and number of yellow follicles in females).

Males demonstrated sensitivity to oxidative stress associated with greater DNA damage in sperm. These data suggest a decrease in sperm quality [45]. Thus, although testicular development and sperm production was not affected, male fertility could be negatively impacted by reduced sperm DNA integrity. One of the major causes of defective sperm function is oxidative stress, which limits the fertilizing potential of these cells [46].

There are many factors which may have an impact on egg production and quality. They range from the bird’s genotype or age [47, 48] to the inclusion of feed additives [49]. However, in this case, it seems that differences in the number of eggs and their quality are secondary effects of sexual maturity (delay of 1 week in puberty). We could hypothesize that birds which start to lay eggs earlier produce more of them than females which achieve maturity later, but our F1-GSE hens always had smaller eggs than controls, even at maximum weight. In mice, during adulthood, female offspring exposed to resveratrol (an antioxidant compound present in grapes) throughout nursing exhibited reduced body weight and increased ovarian weight, but normal oestrous cyclicity [50]. In our study, GSE female offspring laid more eggs and had less broken eggs. Injection *in ovo* of plant extracts (garlic) into Japanese quail eggs improved the laying rate, shell strength and proportion of yolk. This injection modified both the timing of sexual maturity and the quality of the initial eggs [51]. In our case, we found a decrease in egg weight in the GSE group, which was associated with lower fatness around the ovary and a lower fat volume at 37 weeks of age.

Offspring (F2) of the GSE group (F1) were smaller than control (F2) offspring, suggesting a transgenerational effect. The effect of bioactive compounds can change some epigenetic markers. The link between diet-genome interactions, in the nutrigenomics field, was found to inhibit histone deacetylases (HDAC) or DNA methyltransferase (DNMT) activities. Some studies, have screened libraries of natural compounds which possess an epigenetic modifiers activity [52]. In mice, a proanthocyanidin enriched diet decreased the DNMT activity leading to upregulation gene expression induced by hypomethylation of promoter [53, 54]. Few study have investigated the transgenerational effects of maternal bioactive compounds supplementation. Recently, Izquierdo et al., have reported consequence of maternal resveratrol supplementation to offspring and the transgenerational F2 generation, inducing an increase in global DNA methylation in F1 and F2 generation *induced by change of Dnmt3a/b* and *Tet2* gene expression. These results suggest that maternal polyphenol supplementation maintained a phenotype across generations induced by epigenetic transgenerational inheritance mechanism associated to resveratrol exposure [23, 24]. Overall, these recent data raise the hypothesis that grape seed extract diet supplementatioin could exert epigenetic activities leading to trans-generational effects [55]. The more limited growth of F2-GSE chicks can be explained by two hypothetical mechanisms. Firstly, in a low-protein-diet program, this altered both the laying rate and the weight of eggs and chicks that had a lower hatch weight but grew faster after hatching [56, 57]. Authors have shown the transgenerational effect of this type of diet on the growth performance of offspring [58]. Secondly, the rapid development and growth of currently selected bird strains may render the amount of nutrients in the egg insufficient for optimal tissue development [59].

## Conclusion

This study strategy over several generations would be interesting to take into account as part of a feeding strategy for grandparents, which may have an impact on the weight and quality of broilers. This study have shown that maternal nutrition supplemented with GSE had moderate effects on offspring. This supplementation had negative effects; F1-GSE presented a delay in puberty onset and some problems of oxidative stress in muscle. However, it had also positive effects, from an economical point of view, F1-GSE hens laid more eggs, and their eggs were more resistant. A study on the growth of F2 chicks could be interesting to determine if this delay can be caught up and to identify potential epigenetic markers that might be involved in mediating the observed effects.

## Supporting information

S1 Fig Protocol design. The F0 parental hens received a diet enriched with 1% grape seed extract (GSE). Hatching chicks from F0 hens were separated in male and female F1-control and F1-GSE. Body composition and reproductive parameters were analysed. Female F1-control and F1-GSE were fertilized by male controls leading to F2 chick production. The development of F2 chicks was analysed until 12 days of age.
(PDF)

S2 Fig (**A**) Representative picture of computerized tomography analysis of the tibia (red). **(B**) The kinetic of the cortical percentage of the tibia volume was quantified by computerized tomography **(C**) The bone quality was assayed by measurement of the elasticity and bone breaking strength (Newton) of the 37-week-old tibia (n = 20 animals/group). The bone breaking strength was measured using an Instron testing machine (model 5543; Instron S.A., Guyancourt, France). The bone stiffness (slope of the linear part during the flexion test) was also determined. The distance between the two fulcrum points (the length over which mechanical tests were performed) was 6 cm, and the deformation speed was 5 mm/min. Results are presented as means ± SEM. *, p < 0.05. **, p < 0.01.
(PDF)

S1 Table Egg parameters. **(A**) Egg, albumen, yolk and dried shell weights (g), thickness of the shell (mm), and albumen/yolk ratio were measured (n = 60 per group). Eggs were analysed by using an Egg Tester (Egg Tester, Orka Food Technology) with the following parameters: egg resistance (Newton), Haugh unit, Yolk colour between 1 and 16 according to the DSM (formerly Roche), yolk height (mm), yolk diameter (mm), yolk index, volume of albumen (mL) and pH of albumen (n = 70). Results are presented as means ± SEM.
(PDF)
