# Supplementary figures and images for: Maternal dietary supplementation with grape seed extract in reproductive hens increases fertility in females but decreases semen quality in males of the F1 generation
Source: PLoS One. 2021 Feb 25;16(2):e0246750. doi: 10.1371/journal.pone.0246750 (PMC7906403; doi:10.1371/journal.pone.0246750)

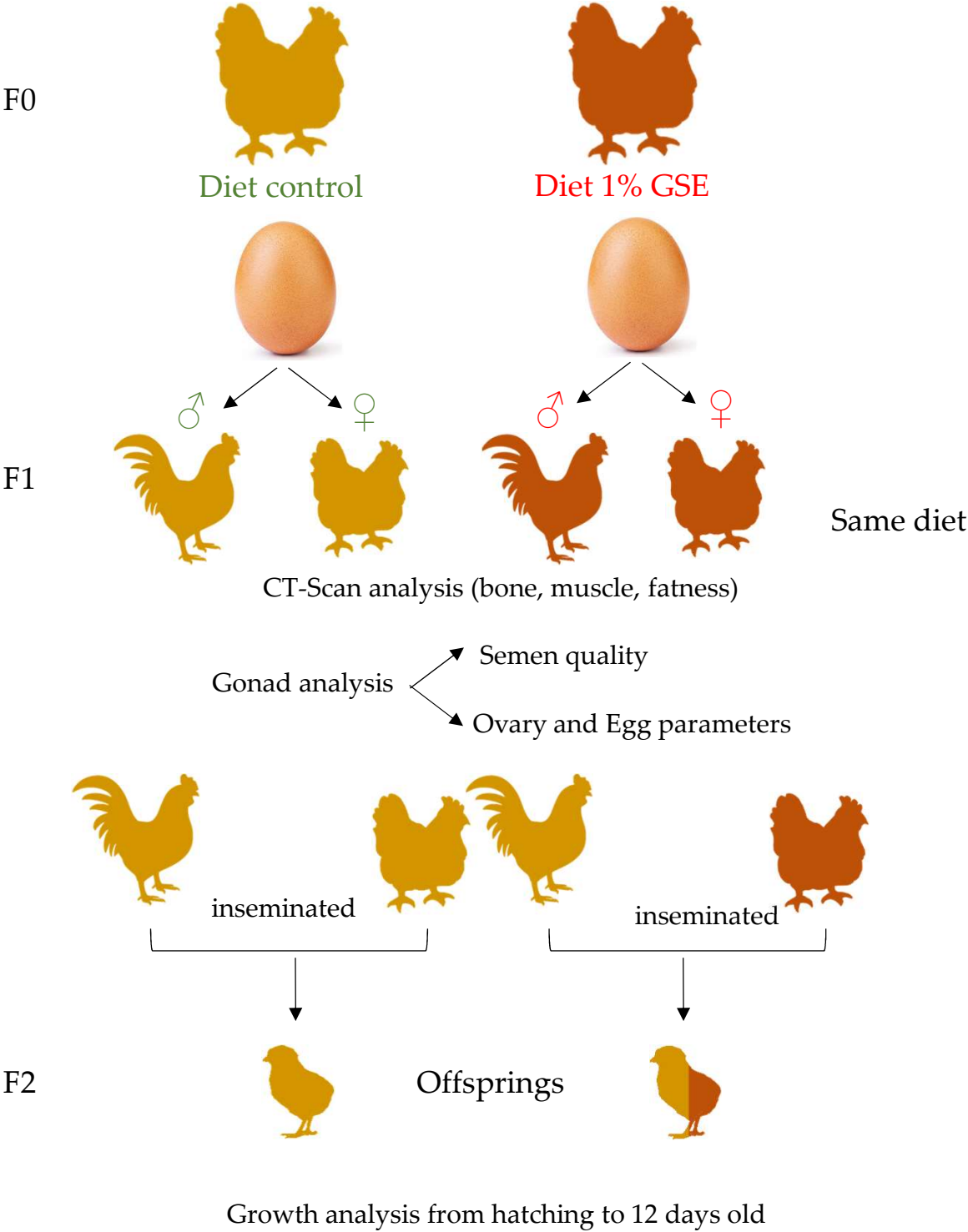

Supplement: S1 Fig — The F0 parental hens received a diet enriched with 1% grape seed extract (GSE). Hatching chicks from F0 hens were separated in male and female F1-control and F1-GSE. Body composition and reproductive parameters were analysed. Female F1-control and F1-GSE were fertilized by male controls leading to F2 chick production. The development of F2 chicks was analysed until 12 days of age. (PDF) [file pone.0246750.s001.pdf]

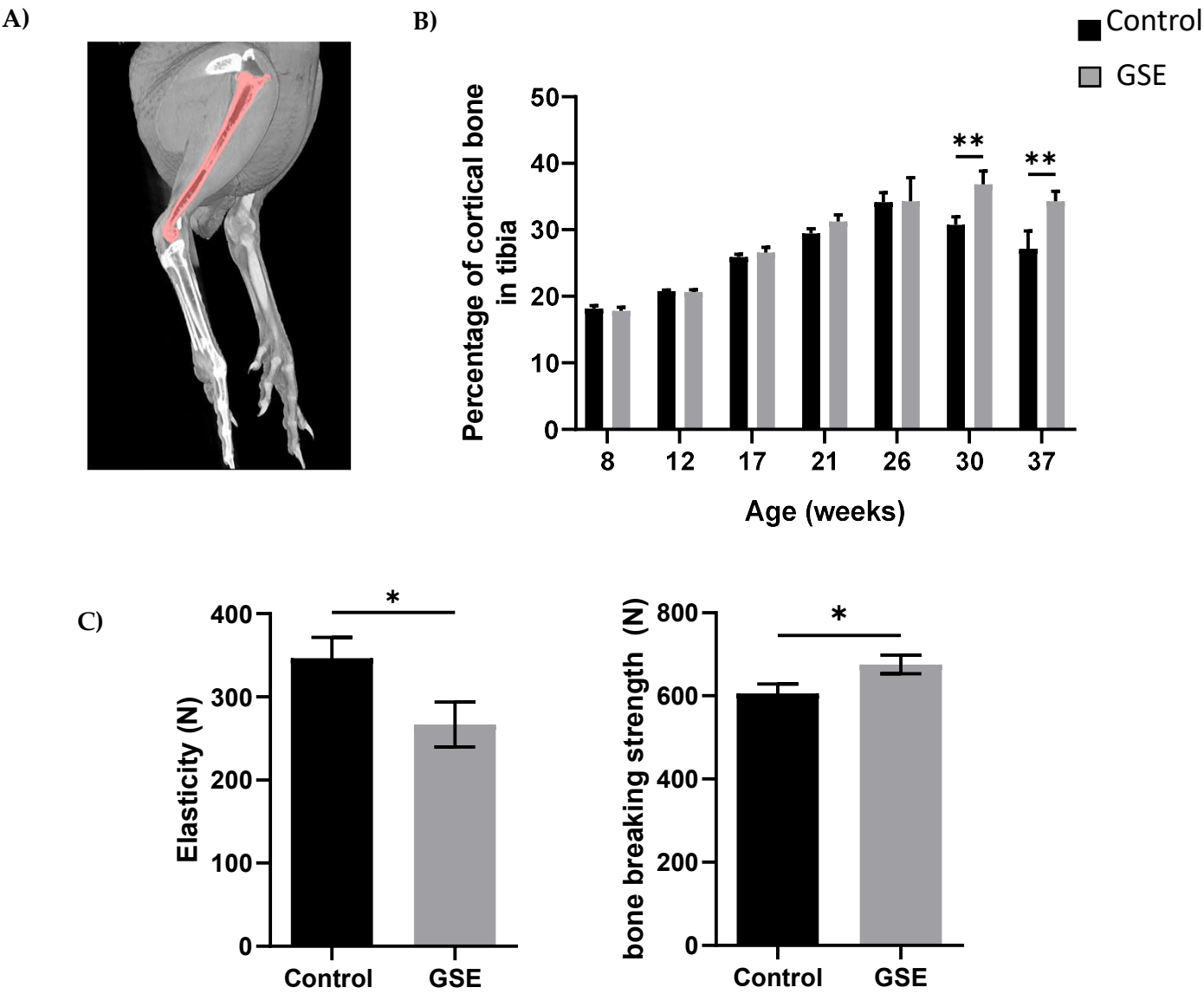

Supplement: S2 Fig — (A) Representative picture of computerized tomography analysis of the tibia (red). (B) The kinetic of the cortical percentage of the tibia volume was quantified by computerized tomography (C) The bone quality was assayed by measurement of the elasticity and bone breaking strength (Newton) of the 37-week-old tibia (n = 20 animals/group). The bone breaking strength was measured using an Instron testing machine (model 5543; Instron S.A., Guyancourt, France). The bone stiffness (slope of the linear part during the flexion test) was also determined. The distance between the two fulcrum points (the length over which mechanical tests were performed) was 6 cm, and the deformation speed was 5 mm/min. Results are presented as means ± SEM. *, p < 0.05. **, p < 0.01. (PDF) [file pone.0246750.s002.pdf]
