# Supplementary material for: Maternal dietary supplementation with grape seed extract in reproductive hens increases fertility in females but decreases semen quality in males of the F1 generation
Source: PLoS One. 2021 Feb 25;16(2):e0246750. doi: 10.1371/journal.pone.0246750 (PMC7906403; doi:10.1371/journal.pone.0246750)
Supplement: S1 Table — (A) Egg, albumen, yolk and dried shell weights (g), thickness of the shell (mm), and albumen/yolk ratio were measured (n = 60 per group). Eggs were analysed by using an Egg Tester (Egg Tester, Orka Food Technology) with the following parameters: egg resistance (Newton), Haugh unit, Yolk colour between 1 and 16 according to the DSM (formerly Roche), yolk height (mm), yolk diameter (mm), yolk index, volume of albumen (mL) and pH of albumen (n = 70). Results are presented as means ± SEM. (PDF) [file pone.0246750.s003.pdf]

S1 Table.

A)

		Egg (g)	Albumen (g)	Yolk (g)	Dried shell (g)	Thickness of the shell (mm)	Albumen/Yolk ratio
Egg from F1-control (n=64)	Mean	60,47	35,10	17,23	6,70	0,37	2,06
	SEM	0,58	0,55	0,28	0,139	0,003	0,04
Egg from F1-GSE (n=46**)**	Mean	59,51	33,46	17,39	7,03	0,37	1,95
	SEM	0,47	0,57	0,31	0,17	0,004	0,05
	P value	**0,0604**	**0,0494**	**0,7222**	**0,1315**	**0,6544**	**0,0856**

B)

		Egg resistance (Newton)	Haugh unit	Yolk color	Yolk height (mm)	Yolk diameter (mm)	Yolk index	Volume of albumen (mL)	pH albumen
Egg from F1-control (n=64)	Mean	37,51	84,30	6,08	19,07	42,41	0,44	34,99	8,39
	SEM	0,9299	0,7432	0,0534	0,0957	0,5763	0,0031	0,4650	0,0666
Egg from F1-GSE (n=46**)**	Mean	37,82	85,03	5,73	18,67	42,98	0,43	34,45	8,07
	SEM	0,9614	0,7447	0,0528	0,1571	0,2512	0,0037	0,3736	0,0922
	P value	**0,8210**	**0,5008**	**<0,0001**	**0,0238**	**0,4258**	**0,0403**	**0,3960**	**0,0055**
